# Supplementary material for: Exploratory study on the psychological impact of COVID-19 on the general Brazilian population
Source: PLoS One. 2021 Feb 3;16(2):e0245868. doi: 10.1371/journal.pone.0245868 (PMC7857630; doi:10.1371/journal.pone.0245868)
Supplement: Attachment — Submitted filename: PONE-D-20-26835 Response to Reviewers.doc [file pone.0245868.s002.doc]

December 21, 2020.

Dear

M. Harvey Brenner, PhD

Academic Editor - PLOS ONE

We appreciate the opportunity to review and resubmit our manuscript PONE-D-20-26835 – “**Exploratory study on the psychological impact of COVID-19 on the general Brazilian population”** in accordance to the reviewers´ suggestions.

In this way, we review the manuscript and respond to all concerns of reviewer 1 and 2. Modifications to the manuscript are highlighted in yellow

**Responses to the Reviewer #1:**

This is, in summary, an interesting paper aimed to explore the prevalence of depression, anxiety, and stress symptoms, and behavioral aspects amidst the COVID-19 pandemic in a Brazilian population. The authors reported that some individuals contracted the virus and had dead friends or relatives. In addition, there was more consumption of drugs, tobacco, medication, and food. Importantly, almost half of participants, especially females, subjects without children, students, patients with chronic diseases, and individuals who had contact with others diagnosed with COVID-19 expressed symptoms of depression, anxiety, and stress.

The authors may find as follows my main comments/suggestions.

**1.** First, when within the Introduction section, the authors correctly reported that isolation and quarantine linked to Covid-19 infection are associated with psychological problems such as anxiety, depression, sleep disorders, abstinence, regression, and hallucinations, they could even briefly describe the involvement of sensory perception which is implicated in emotional processes of individuals with psychological problems. Importantly, the unique sensory processing patterns in subjects psychological problems have been reported. Hyposensitivity or hypersensitivity may be "trait" markers of individuals with anxiety and depression and interventions should refer to the individual unique sensory profiles and their behavioral and functional impact in the context of real life. Thus, given the above information, my suggestion is to include throughout the manuscript, the paper published in 2016 on Psychiatry Res (PMID: 26738981).

**Authors’ response:** Suggestion included

**2.** In addition, the authors might even report the link between anxiety, depression, and specific adverse clinical outcomes (e.g., suicidal behavior). Importantly, depressed patients may frequently display affective dysregulated temperaments and suicidal behavior. Overall, more than half of patients with dysregulated temperaments reported higher levels of hopelessness and may be considered at suicide risk. Thus, in order to briefly address the mentioned topics (although i understand that the link between anxiety, depression, and negative outcomes including suicidal behavior) is not the main aim of investigation of the present manuscript, the study below (PMID: 23104655) could be included throughout the main text by the authors.

**Authors’ response:** Suggestion included

**3.** Moreover, as the most relevant aims/objectives of the present study have been directly reported throughout the Introduction section, the main study hypotheses should be similarly described within the main text.

**Authors’ response:** Done

**4.** Furthermore, as inclusion criteria have been well described, exclusion criteria need to be extensively reported.

**Authors’ response:** Exclusion criteria included forms sent after June 5, 2020. Thus, thirty-one participants were excluded once they were sent the form after the deadline.

**5.** Furthermore, more information about the online questionnaire comprising 108 questions could be provided for the general readership.

**Authors’ response:** More details have been provided.

**6.** In addition, the authors should immediately present and discuss, in the first lines of the Discussion section, their most relevant study findings. Conversely, they seem to focus with redundance on the main introductive statements as well as upon the main aims/objectives of this paper that should be stressed elsewhere within the main text.

**Authors’ response:** Done

**7.** Finally, which type of interventions the authors suggested for subgroups of subjects who were found to be more susceptible to the psychological impact of Covid-19 infection? Here, more details/ information are required to this specific regard.

**Authors’ response:** Suggestions have been provided. (expanding psychiatric care services, training more qualified professionals in the management of psychological impacts in the face of the pandemic, within the scope of cognitive-behavioral therapy and psychoeducation, for example, will certainly reduce the mental health problems in this population)

**Responses to the Reviewer #2:**

Dears author,

The review of the article allows us to verify the commitment and the accuracy of the authors. So this review is intended to indicate some minor modifications listed below:

**1-** I suggest the revision of the central hypothesis presented at the end of the introduction. Although the authors compare the findings with other studies, the hypothesis does not highlight the main findings of the present study.

**Authors’ response:** Central hypothesis revised.

**2 -** It is unclear whether the authors make use of guidelines for scientific disclosure and transparency. Considering the methodological design and the way that the data were obtained, the use of these guidelines is encouraged (eg CHERRIES, STROBE ...). Therefore, authors can check out The EQUATOR Network.

**Authors’ response:** The methodological design and the way that the data were obtained have been disclosed in the method section.

**3 -** it is unclear if there were any leaks to the sample.

**Authors’ response:** There was no leaks to the sample.

**4 -** it is unclear whether there are any missing in the data, and how these were considered for statistical analysis.

**Authors’ response:** There was no missing data.

**5 -** I suggest improving the information regarding the socio-demographic questionnaire. With more specificity on questions and answer categories.

**Authors’ response:** Information provided in measure section.

**6 -** It is not clear how the authors made the adaptation of the "Coping Strategy Inventory" and the criteria used by them.

**Authors’ response:** Clarified information in the method section.

**7 -** The last paragraph of item 2.1 would be better allocated in item 2.3.

**Authors’ response:** Information allocated.

**8 -** In the ‘data analysis’, it is not clear whether the significance level adopted concerns only chi-square or all statistical test.

**Authors’ response:** The p-value <0.05 was considered significant in this study for all statistical analysis.

**9 -** It is encouraged to refer to the literature, regarding the parameters adopted for the interpretation of the Rho.

**Authors’ response:** Dancey CP, Reidy J. Statistics without maths for psychology. 5th ed. Harlow: Prentice Hall. 2011.

**10 -** The criteria used for dividing the participants into age groups are not clear.

**Authors’ response:** The criteria used for dividing the participants into age groups was based on Brazilian study by Pinheiro et al. (2020) which analyzed stress during the COVID-19 pandemic in a situation of social distance.

(Pinheiro GA, Luna GI, Santos RAC, Pimentel SFP, Varão AC. Perceived stress during a period of social distancing: differences between sexes. Braz. J. Hea. Rev. 2020; 3(4):10470-10486. https://doi.org/10.34119/bjhrv3n4-264)

**11-** Table 1 reports data that were not used in other statistical analyzes and that contribute little to the discussion of the study. Authors are invited to verify the need for these data in the article.

**Authors’ response:** It has been excluded data not used in the statistical analyzes and with little contribution

**12 -** The inclusion of subtitles in table 2 is suggested, regarding the statistical test used to provide the p value.

**Authors’ response:** The *p* value was provided by using chi-square statistical test.

**13 -** In table 3, the mean and standard deviation values were provided. However, considering the non-parametric analyzes, the inclusion of the median, respective CI or Interquartile Range, plus the percentage of the frequency for the chi-square test is suggested.

**Authors’ response:** Median, CI, and percentage provided in the tables 3, 4, and 5.
